# Encapsulation of *Saccharomyces boulardii* into sugar pellets coated with an enteric polymer

**DOI:** 10.1007/s11274-025-04649-4

**Published:** 2025-10-30

**Authors:** Karla Lilian Bailon-Barceinas, Zacnite Sánchez-Portilla, Alma Delia Román-Gutiérrez, Luz María Melgoza-Contreras, Raquel González-Vázquez, Lino Mayorga-Reyes, María Angélica Gutiérrez-Nava

**Affiliations:** 1 Maestria en Ciencias Farmaceuticas, Departamento de Sistemas Biologicos, Universidad Autonoma Metropolitana, Unidad Xochimilco, Ciudad de Mexico, 04960 Mexico; 2 Doctorado en Ciencias Biologicas y de la Salud, Universidad Autonoma Metropolitana, Unidad Xochimilco, Ciudad de Mexico, 04960 Mexico; 3 Area Academica de Quimica, Universidad Autonoma del Estado de Hidalgo, Ciudad del Conocimiento, Carretera Pachuca- Tulancingo Km 4.5s/n, Mineral de la Reforma, Hidalgo, 42184 Mexico; 4 Laboratorio de Farmacotecnia, Departamento de Sistemas Biologicos, Universidad Autonoma Metropolitana, Unidad Xochimilco, Ciudad de Mexico, 04960 Mexico; 5 Laboratorio de Biotecnologia, Departamento de Sistemas Biologicos, SECIHTI-Universidad Autonoma Metropolitana, Unidad Xochimilco, Ciudad de Mexico, 04960 Mexico; 6 Laboratorio de Biotecnologia, Departamento de Sistemas Biologicos, Universidad Autonoma Metropolitana, Unidad Xochimilco, Ciudad de Mexico, 04960 Mexico; 7 Laboratorio de Ecologia Microbiana, Departamento de Sistemas Biologicos, Universidad Autonoma Metropolitana, Unidad Xochimilco, Ciudad de Mexico, 04960 Mexico

**Keywords:** Encapsulation, Eudragit ^®^ S12.5, Intestinal delivery, Modified release system, Saccharomyces boulardii

## Abstract

Many current probiotic products do not optimally release enough viable cells at the site of action due to the loss of viability during manufacturing and passage through the stomach. This work aimed to formulate a modified release system that protects *S. boulardii* during the manufacturing process and under simulated acidic gastric conditions. *S. boulardii* was layered to sugar pellets, coated at 5% and 10% with the enteric polymer Eudragit^®^ S12.5, and encapsulated in hard gelatin capsules. During manufacturing, the main determinant of viability was the incorporation of yeast onto the sugar pellets. The viability *S. boulardii* in the pellets unprotected was 87.7% ± 1% after 80 min, whereas coating of unprotected pellets with 5% polymer required 54 additional min (total: 134 min) and the viability was 92.6% ± 1%, while 10% coating took 55 additional min (total: 189 min) and viability was 88.7% ± 1.8%. Notably, *S. boulardii* coated pellets were prepared before the recovery process. The weight variations of the pellets contained in the capsules were 0.53 ± 0.032 g and 0.55 ± 0.029 g for 5% and 10% coating, respectively. The disintegration time for each formulation was 18 min, which is below the limit of Mexican and European Pharmacopeias (30 min respectively). In the dissolution test, no release occurred under acid pH, regardless of the Eudragit percentage. In contrast, under alkaline pH conditions, polymer dissolution occurred, releasing *S. boulardii* 65% ± 0.46% and 68% ± 0.23% for the 5% and 10% enteric coatings, respectively, after 20 min in alkaline condition the release was 81% ± 0.48% and 87% ± 0.33% respectively. In conclusion, this modified-release system may serve as an alternative for preserving the viability of *S. boulardii* during gastric passage, potentially increasing the number of viable yeast cells that reach the site of action, primarily the small intestine.

## Introduction

Probiotics are “live microorganism that, when administered in adequate amounts, confer a health benefit on the host” (Liu et al. 2023). For several decades, studies have demonstrated their significant health benefits, leading to their widespread use in the scientific community, pharmaceutical, and food industries (Chen 2021; Latif et al. 2023; Roy and Dhaneshwar 2023; Suez et al. 2019). Despite this, a major challenge is the loss of viability that occurs during manufacturing processes and storage due to factors like temperature, oxygen, process time, and humidity (De Simone 2019; Sionek et al. 2024; Wang and Zhong 2024). When ingested, probiotics face an even greater threat from gastric acidity, bile salts, and digestive enzymes, with studies showing that up to 60% of probiotics can perish in the gastric environment before reaching their site of action (Castro-López et al. 2023).

Given the growing evidence of probiotic benefits, there is a strong interest in developing stable solid products with proper functional characteristics, such as resistance to temperature and pH fluctuations. These products could be added to oral solid dosage forms or dairy products to ensure the microorganism are released directly at their site of action. To this end, researchers have explored various tools to ensure the viability of probiotics during processing, storage, and passage through the gastrointestinal tract (Mudgil et al. 2024; Thinkohkaew et al. 2024). Probiotic encapsulation techniques, which use protective materials as matrices to physically retain the microorganism and allow for controlled release, have been particularly successful (Vergkizi et al. 2024). Encapsulation with enteric polymer coatings has emerged as a particularly advanced strategy to enhance probiotic viability and ensure efficacy.

Enteric coating systems are designed to protect dosage forms during gastric transit and control the release of the active ingredient in the intestine. These systems have been applied to both monolithic dosage units and multiunit pellet systems (Majeed et al. 2020; Yus et al. 2019). Dual layer core encapsulation systems, for instance, using alginate followed by a coating with Eudragit^®^ L100-55 or S100 offers targeted release and protects from the gastric environment (Razavi et al. 2021; Xu et al. 2025). Studies have shown marked improvements with these materials; for example, alginate tapioca microspheres coated with Eudragit^®^ L100-55 demonstrated a survival rate of approximately 90% for *L. plantarum* after 120 min under simulated gastrointestinal conditions (Łętocha et al. 2024). Similarly, a study using *L. rhamnosus* GG spray-dried with Eudragit^®^ L100-trehalose reported a high survival rate (87–93%) after in vitro digestion (Xu et al. 2025). Other research confirms that polymethacrylate enteric coating like Eudragit^®^ L100-55 and S100 remain robust in acidic conditions and dissolve at intestinal pH, providing effective controlled release without compromising viability (Ahuja 2024; Nikam et al. 2023).

Probiotics are often administered in capsules or tablets, but these dosage forms may not be suitable for individuals with swallowing difficulties, such as children and the elderly (How and Yeo 2021; Kandur et al. 2024; Wang et al. 2022). Alternatively, formats like powders or granulates, can be more easily consumed by these group (Baral et al. 2021; Jacobsen et al. 2021).

*S. boulardii*, specifically, is widely used as an antidiarrheal agent and for treating various gastrointestinal disorders (Kelesidis and Pothoulakis 2012; Tung et al. 2009). However, it is highly sensitive to acid conditions, which can compromise its survival through the stomach (Carvalho Bruna et al. 2024). Because the health benefits of probiotics are directly linked to the number of viable cells that reach the intestine, technological innovations are needed to enhance the viability of *S. boulardii* and improve its therapeutic efficacy. One such strategy is the incorporation of probiotics into a modified-release system with enteric polymers. In a previous study, we developed a powdered product with an enteric coating for *Bifidobacterium* spp. using commercial microcrystalline cellulose and prebiotic inulin as cores (Sánchez-Portilla et al. 2020) demonstrating a successful approach for protecting a probiotic.

This study proposes a modified-release system to address the specific challenge of protecting the viability of *S. boulardii.* The use of enteric coatings has not yet been reported for this specific probiotic. Our aim was to formulate a modified release system that protects *S. boulardii* during the manufacturing process and under simulated acidic gastric conditions. We accomplished this by incorporating viable *S. boulardii* cells onto the surface of sugar pellets and coating them with an enteric polymer at 5% and 10% concentrations, selected based on their proven effectiveness in previous studies (Sánchez-Portilla et al. 2020; Yus et al. 2019). We assessed cell viability throughout the manufacturing process to ensure biological integrity, and evaluated capsule uniformity of weight, disintegration time, and dissolution profiles. By protecting *S. boulardii* during both processing and gastric passage, this system has the potential to significantly increase the number of viable cells that reach the small intestine, thus enhancing its therapeutic efficacy.

Cell viability was assessed along the manufacturing process to ensure biological integrity. Subsequently, capsule uniformity of weight, disintegration time and dissolution profiles were evaluated. The 5 and 10% polymer concentrations were selected based on previous studies demonstrating their effectiveness in protecting probiotics and enabling controlled release in the gastrointestinal tract.

## Materials and methods

### Microorganisms and culture conditions

*S. boulardii* was obtained via the inoculation of a commercial lyophilizate (Floratil, Merck) in YPD media (glucose and meat peptone 2%, yeast extract 1%) and incubation at 37 °C at 200 rpm under aerobic conditions. The initial CFU of the culture was 11.6 log_10_. The strain is currently part of the collection from the Microbial Ecology Laboratory of Universidad Autonoma Metropolitana campus Xochimilco.

### Manufacturing process to encapsulate *S. boulardii*

#### Layered coating process of *S. boulardii* into sugar pellets

**Fig. 1:**
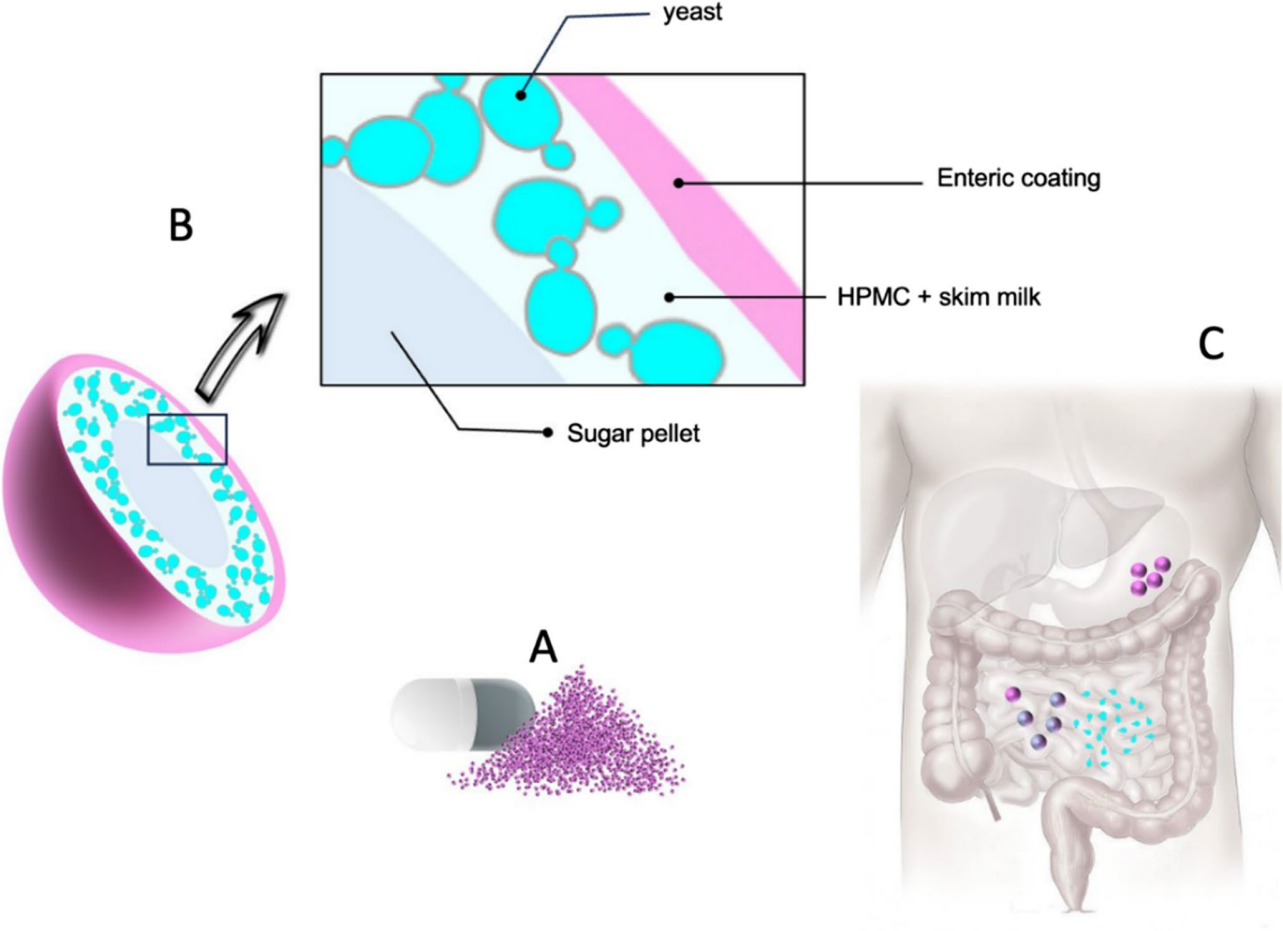
Schematic representation of **a**) pellets filled into hard gelatin capsules, **b**) internal structure and **c**) the specific release site along the small intestine

Layered coated technique was employed throughout the entire process to obtain pellets containing yeast to subsequently coated them with Eudragit^®^ S12.5 polymer. An aliquot of yeast was activated by three consecutive passages in 250 mL of YPD liquid medium. The cultures were incubated at 37 °C with constant shaking (200 rpm) for 24 h. After that, the cell pellet was obtained through two washes cycles (8,000 rpm/10 min and 3,000 rpm/15 min). Resulting cell pellet was suspended by gently shaking 150 mL of a mixture of 30% skim milk and 2% HPMC (hydroxypropylmethylcellulose) in physiological saline solution previously integrated by mechanical stirring (Caframo BDC2002). This suspension was used to coat sugar pellets of 1.0 to 1.19 mm in diameter (M16/18 Colorcon^®^) by using the film recovery technique in a conventional pump. The conditions used during the process were as follows: rotation speed of 17–23 rpm, spraying speed of 3–4 rpm, temperature of 37 °C, and atomization pressure of 20–25 psi. After the process, a sample was taken to analyze the viability of the yeast via plate counting. Subsequently, the pellets containing the incorporated yeast were coated with the Eudragit^®^ S12.5 polymer via the same film coating technique. The polymer was prepared according to the manufacture´s specifications by using isopropanol as dissolvent. Two weight gains (wg) of 5% and 10% were used, resulting in an excess of 2% for both cases. The conditions used during this process were as follows: rotation speed, 17–23 rpm; spraying speed, 15–17 rpm; temperature, 30–33 °C; and atomization pressure, 20–25 psi. Finally, a sample was taken to analyze the viability of the yeasts. All the experiments were performed in triplicate. Figure 1 displays the pellets filled into hard gelatin capsules, alongside a schematic representation of their structural composition and the targeted release site within the gastrointestinal tract (Bailon Barceinas 2020; Sánchez-Portilla et al. 2020).

#### Determination of viable yeasts

One gram of biomass of *S. boulardii* without enteric protection or pellets formed with enteric coating were dissolved in 50 mL of phosphate buffer (pH 7.5). In both cases, 100 µL of sample was taken, and serial decimal dilutions were carried out with YPD culture medium. A total of 100 µL of sample was seeded in triplicate on solid medium (YPD) via the rod extension technique, and subsequently, the boxes were incubated for 72 h at 37 °C. Finally, the colony forming units (CFUs) were enumerated, and the results were expressed as log_10_ CFU (Blaize et al. 2019). The encapsulation efficiency was determined by using the following formula 1. This efficiency was calculated (Ganje et al. 2024) considering the initial CFU count of 11.6 log_10_.

1 $$\begin{array}{c}\begin{array}{c}\begin{array}{c}Encapuslation\:efficiency\:\left(\%\right)=\\\frac{After\:5\:or\:10\:\%\:of\:coating\:{CFU\:\log}_{10}}{Initial\:CFU\:\log_{10}}\times100\end{array}\end{array}\end{array}$$

#### Hard gelatin capsules acquisition

The hard gelatin capsules number 0 were filled with pellets with and without enteric protection via a Profiller^®^ 1100 manual encapsulator (USA). The determination of the dose per capsule was carried out by establishing the quantity of viable yeast per 1 g of pellets contained in a capsule. All the experiments were performed in triplicate.

### Physical characterization of the capsules

#### Weight uniformity of capsules containing coated pellets

This test was conducted according to the general method of analysis (GMA) 0571 from *Pharmacopoeia of the United Mexican States* FEUM 11th edition (2014). The procedure was carried out as follows. A total of 20 capsules were individually selected at random from the batch under study for analysis. Each intact capsule was weighed using an analytical balance (PA214C, Ohaus corporation, USA) with a precision of ± 0.1 mg. All weighing were conducted under controlled environmental conditions (temperature: 20–25 °C, relative humidity: 40–60%, and minimal vibration). The total weight was recorded for each capsule. The capsules were carefully opened, and their contents were completely removed. The empty shells were then weighed individually. All residues were removed to ensure accurate determination of the capsule shell weight. The net content weight for each capsule was obtained by subtracting the empty capsule weight from the total weight. The average net content was calculated from the 20 units. According to the acceptance criteria, no more than two capsules were allowed to deviate from ± 10% of the average net weight, and none were allowed to deviate beyond ± 20%.

#### Disintegration of capsules

Disintegration was assessed following the GMA 02611 established in the FEUM 11th edition (2014). The limits used for comparison was the ones stablished in FEUM 11th edition (2014) and European Pharmacopeia 11th edition (2023).

This method evaluates, the time required for a solid pharmaceutical form to disintegrate in a test fluid within a defined period and under specific operating conditions. It is important to note that disintegration does not imply complete solubilization of the gelatin capsule or its contents, nor of the tablet itself. Rather, it refers to the condition in which only insoluble fragments, coating residues, or gelatin remnants remain on the mesh of the apparatus.

The assay involved the use of a basket which constituted the main component of the apparatus and consisted of a rigid assembly designed to support six cylindrical glass tubes. Each tube measured 77.5 ± 2.5 mm in length and had an internal diameter of 21.5 mm. The wall thickness was approximately 2 mm. The tubes were maintained in vertical position by their attachment to two superimpose, transparent plastic plates, each measuring 88 to 92 mm in diameter and 5 to 8 mm in thickness. These plates were perforated with six evenly spaced holes, equidistant from the center, serving to support the tubes. At the bottom of each of the six holes in the lower plate (referred to as the grid), a stainless-steel mesh screen was fixed. The mesh was made of wire with a diameter between 0.600 and 0.655 mm and corresponded to mesh number 10 (with an opening size of 1.8 to 2.2 mm). To ensure the vertical alignment of the glass tubes, the plastic plates were held parallel by a central stainless-steel rod approximately 180 mm in length. The upper end of this rod featured a slot that allowed the basket to be mounted onto a mechanical device. This device was designed to produce a regular, vertical reciprocating motion with no appreciable horizontal deviation, having an amplitude of 53 to 57 mm. The number of complete up and down displacements of the basket ranged from 28 to 32 cycles per minute.

A capsule was placed in each of the six glass tubes of the basket, and a removable wire mesh screen (mesh No.10) was positioned on the upper part of the basket. The basket was then immersed in a glass vessel containing water at 37 ± 2 °C. The vessel measured between 139 and 155 mm in height, with an internal diameter ranging from 97 to 110 mm. The capsules were observed until disintegration occurred. At that point, the basket was lifted to separate the immersion medium from the disintegrated capsule residues, and the disintegration time was recorded. All experiments were performed in triplicate.

#### Capsule dissolution profile

To evaluate the release of *S. boulardii* from the coated pellets contained in the capsule under acidic and alkaline conditions, the methodology established by the FEUM, 11th edition (2014), method MGA 0521 for oral delayed-release system (general method B) was followed. Dissolution apparatus I was employed, in which the capsules were subjected to an acid phase using 500 mL of 0.1 N HCl pH 1.2 and maintained at 37 ± 0.5 °C for 60 min under constant stirring at 100 rpm. Aliquots of 4 mL were collected at 0, 30, and 60 min.

After completing the acid phase, the remaining volume of HCl containing the capsules were centrifuged, then the supernatant was decanted. Any remaining HCl was carefully removed using a pipette. To continue with the experiment, the pellet was placed in phosphate buffer at pH 7.5, homogenized, and the pH determined to ensure that it was not changed. In this step, the dissolution apparatus was operated for 20 min to ensure total disintegration of the pellets. During this phase, 4 mL aliquots were taken at 0, 10 and 20 min. To determine the viability of the samples obtained in the acidic medium, a neutralization process was carried out using serial dilutions with sterile saline solution (SSF) and YPD culture medium, to counteract the effects of pH during microbiological incubation. The viability of each sample was assessed according to the previously described procedure. All experiments were performed in triplicate.

### Statistical analysis

To determine significant differences (*p*≤ 0.05) in the viability of *S. boulardii* during the spraying process onto sugar pellets after applying enteric protection and performing disintegration, weight variation, and dissolution profile test and ANOVA was conducted using GraphPad Prism software.

## Results

### Viability of *S. boulardii* during the manufacturing of pellets without and with enteric protection

**Fig. 2:**
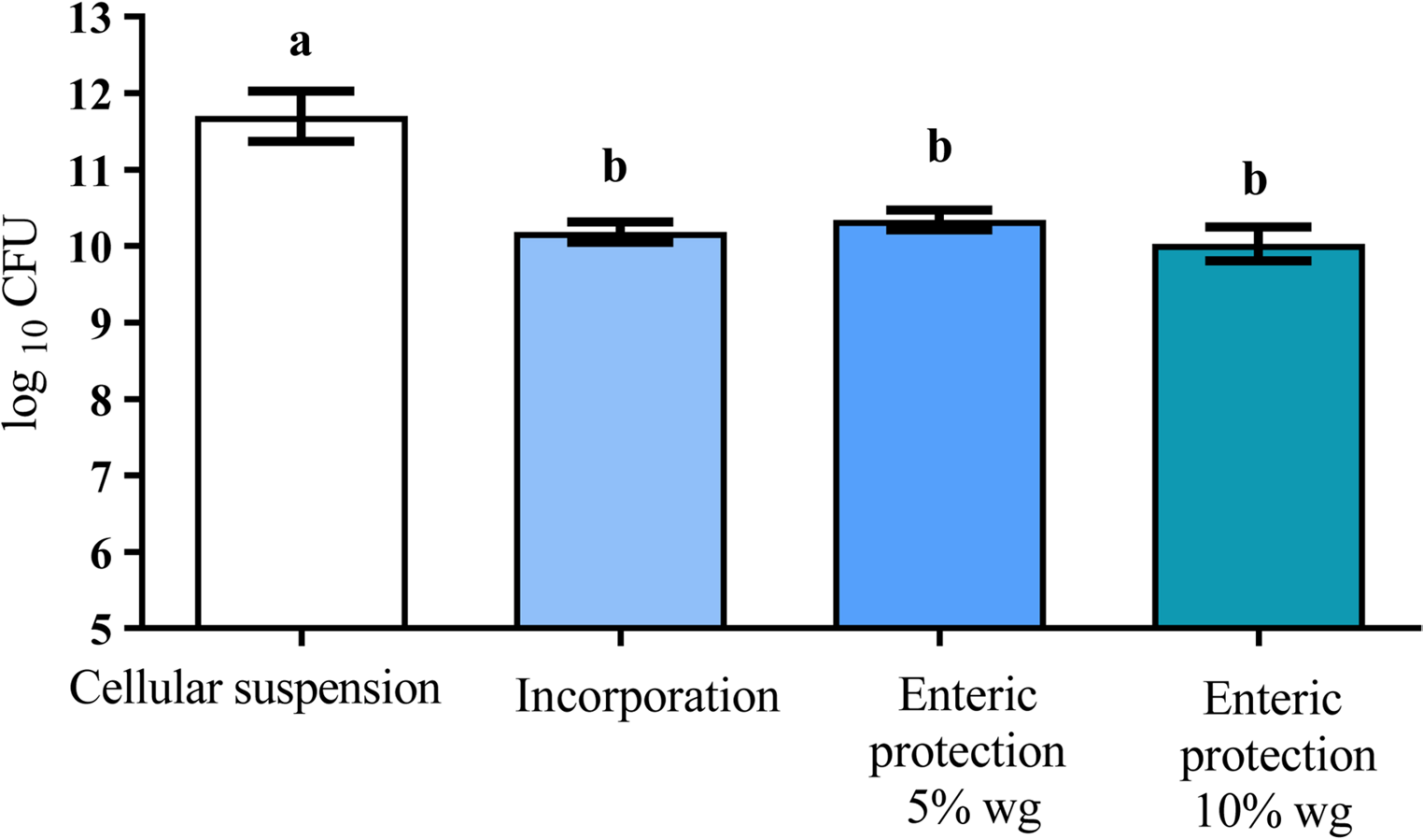
Viability of *S. boulardii* during its incorporation onto the sugar pellets and after enteric coating. Each bar represents the mean of three independent trials, along with the corresponding standard deviation. A significant loss of viability was observed. Different letters mean significant differences (*p* < 0.0001)

During the process of incorporating the yeast over the sugar pellets (10.1 ± 0.13 log_10_ CFU), there was a significative decrease (*p* < 0.0001) of 1.5 log_10_ CFU with respect to the amount of yeast in the initial culture (11.6 ± 0.33 log_10_), which was opposite that of the enteric protection processes (5% and 10% wg) since it remained on the same order of magnitude (10.3 ± 0.13 and 10.0 ± 0.22 log_10_, respectively). Significant differences (*p* < 0.0001) were also found between initial count of the cellular suspension, incorporation and 5 and 10% of enteric protection (Fig. 2).

The process of generating pellets without enteric protection took 80 min. The enteric coating process using 5% polymer required 54 min; thus, the total time to produce pellets coated at 5% was 134 min. To achieve a 10% of coating, an additional 5% polymer was applied, which took another 55 min. Therefore, the total time to produce pellets coated at 10% was 189 min. The encapsulation efficiency was higher to 5 than 10% of enteric protection (Table 1). No significant differences were found. It is worth nothing that the pellets coated with *S. boulardii* were prepared prior to the recovery process.

**Table 1:** Viability, processing times and yields of *S. boulardii* pellets with 5% and 10% enteric protection and without protection. Results represents the average of three independent trials, along with the corresponding standard deviation

Process	Viability (%)	Total time of the process (min)	Yield of enteric protection (%)	Encapsulation efficiency (%)
No protection	87.7 ± 1	80		
5%	92.6 ± 1	134	100	89 ± 1
10%	88.7 ± 1.8	189	99.61	86 ± 1.8

In all experiments no significant differences were found (*p* ≥ 0.05)

In addition, the results show that the process of coating did not impact over the percentage of viability and that the process of recovery was efficient, since 100% of pellets were coated with 5% polymer, and 99.61% with 10% polymer. Regarding the observed differences between the 5% and 10% concentrations, it is important to highlight that even small variations in polymer concentration can influence the structural integrity of the microcapsules and the release kinetics of the probiotic. Moreover, higher concentrations may increase particle size, which could negatively affect the bioavailability and acceptability of the final product (Erdélyi et al. 2022). Therefore, the selection of these concentrations was based on a balance between the effectiveness of probiotic protection, cell viability, and the desired physicochemical properties of the delivery system.

### Physical characterization of the capsules

The determination of the uniformity of weight and disintegration time of the capsules obtained was carried out as part of the quality control of the finished product. The results are shown in Table 2. According to the results, the capsules containing pellets coated with 5% and 10% of enteric protection exhibited a percentage variation of less than ± 10%. No significant differences were found between capsules protected with 5% and 10% of enteric coating.

**Table 2:** Quality control of the obtained capsules

Test	Capsules	Capsules	FEUM	EP
	5% wg	10% wg	specifications	
**Weight variation**	**a.** 0.53 g ± 0.032 **b.** 6.10%	**a.** 0.55 g ± 0.029 **b.** 5.35%	A maximum of 2 of the 20 capsules deviated within ± 10% of the average weight, none deviated more than ± 20%.	No more than 2 of the 20 capsules deviated from ± 10% of the average net content, and none deviated from ± 20%
**Disintegration**	18 min	18 min	30 min as limit	30 min as limit time

wg: weight gain. a: Average value and ± standard deviation. b: coefficient of variation. NA: does not apply. FEUM: Pharmacopoeia of the United Mexican States 11th edition (2024). EP: European Pharmacopeia 11th edition (2023). In all experiments no significant differences were found (*p* ≥ 0.05)

**Fig. 3:**
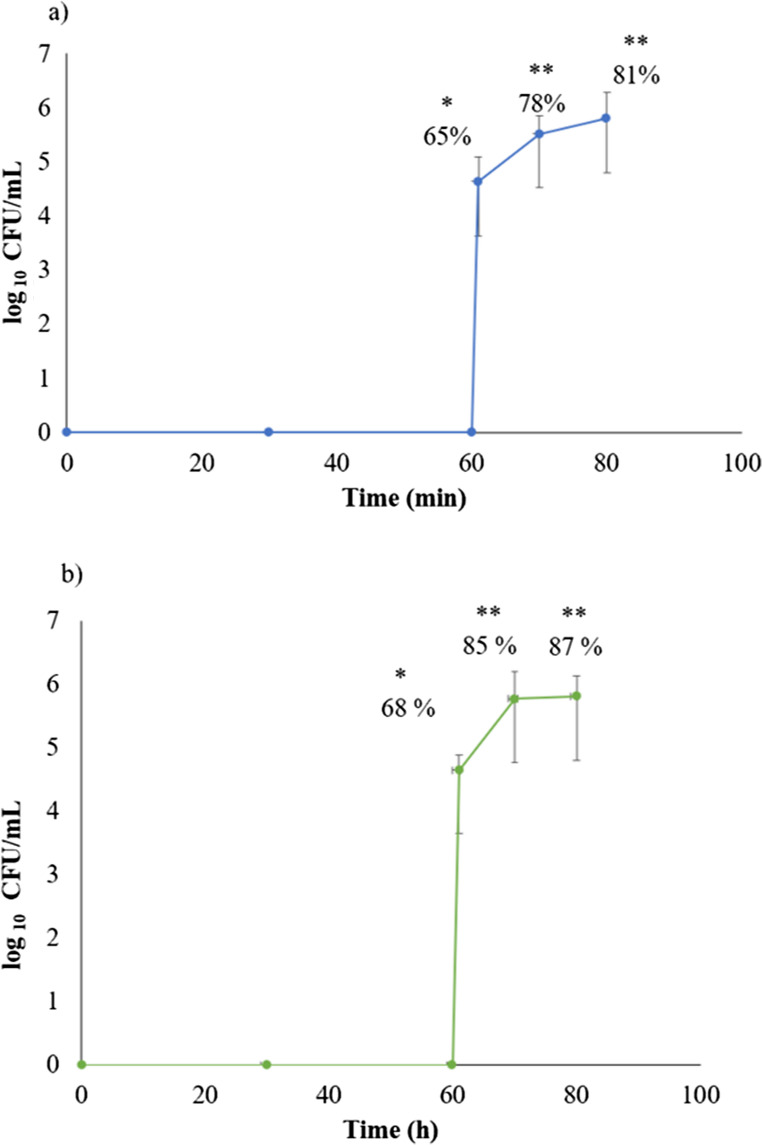
Dissolution profile of capsules with **a**) 5% and **b**) 10% weight gain from the enteric polymer. Each point represents the mean of three independent trials, along with the corresponding standard deviation. The percentage of release is shown at the different time points evaluated. * vs. ** indicate significant differences (*p* < 0.0001)

In the acidic media, at 5% and 10% coating there was no release of the yeast, during the 60 min of evaluation. However, after changing to basic medium (Fig. 3), the polymer started to dissolve, with probiotic release of 65% ± 0.46% and 68% ± 0.23% for the 5% and 10% enteric protection formulations, respectively. After ~ 20 min at basic pH, total dissolution occurred, with probiotic release rates of 81% ± 0.48% and 87% ± 0.33%, respectively, for both percentages of enteric protection (Fig. 3). The statistical analysis revealed no significant difference (*p* ≥ 0.05) between the samples with different percentages of polymer weight over an average time of 20 min (Fig. 3). However, there was significant difference (*p* ≤ 0.05) between the initial time of release (60 min) and 10 min after initial release to both percentages of covering. After this time, release is constant along the time (Fig. 3).

**Fig. 4:**
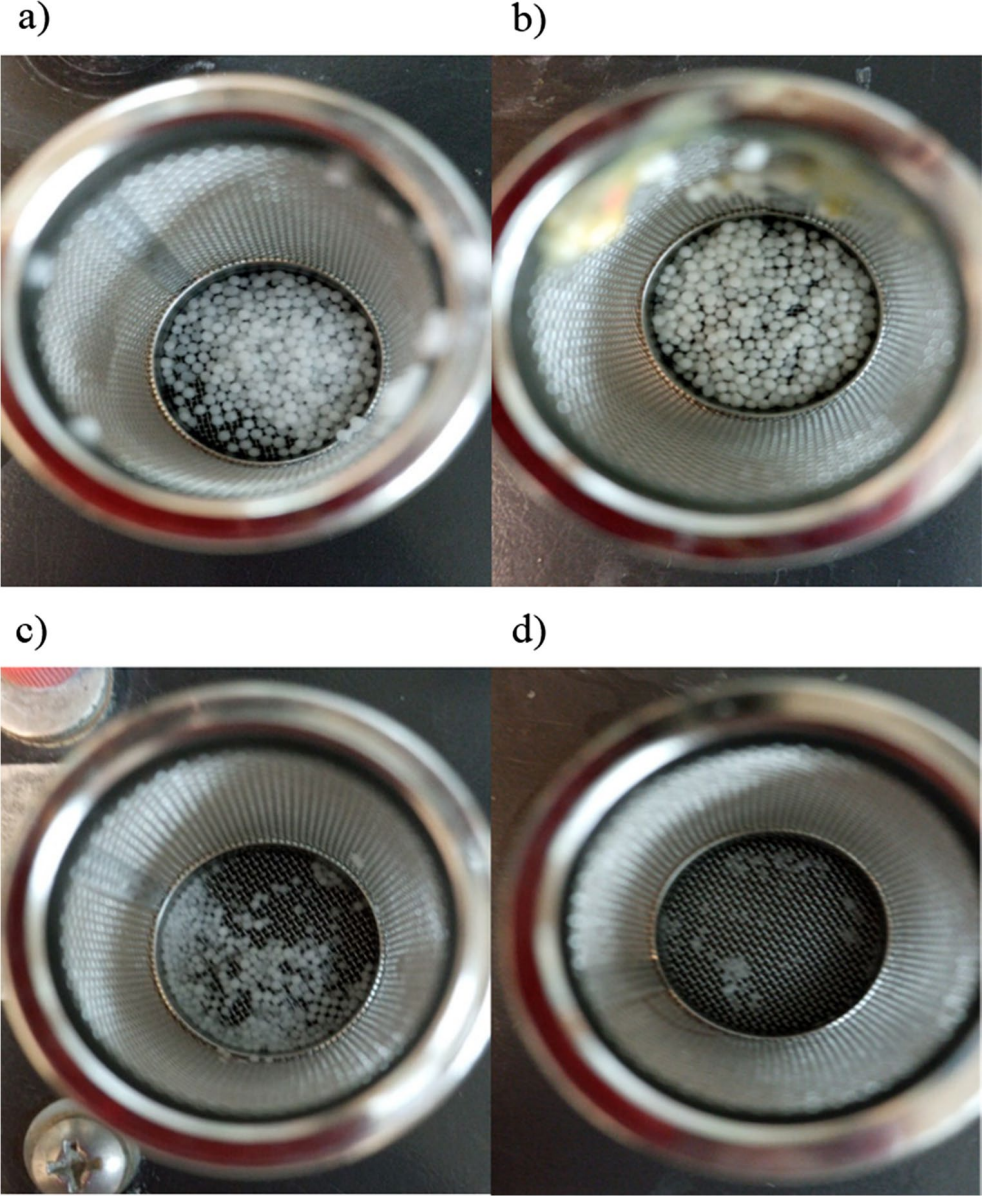
Physical appearance of the pellets during dissolution in acidic and alkaline media. **a**) and **b**) intact pellets at 5% and 10% wg enteric protection, respectively, after hard gelatin capsules were disintegrated in acidic media. **c**) and **d**) disintegrated pellets at 5% and 10% wg enteric protection, respectively, after the pellets were in basic pH solution

The physical appearance of the pellets during the dissolution test is shown in Fig. 4, where (a) and (b) show the intact pellets at 5% and 10% wg enteric protection after the hard gelatin capsules were disintegrated in acid medium.

In acidic media, the size or shape of the pellets did not change due to the enteric protection conferred; in contrast, in alkaline media, the size or shape of the pellets did change (Fig. 4c and d). Disintegration of the pellets is observed because of the loss of integrity of the enteric coating.

## Discussion

The results suggest that the film coating technique applied to the core was effective in maintaining yeast viability following exposure to stressors such as temperature and drying pressure. The polymeric layers formed, along with HPMC and skim milk in the incorporation mixture, likely contributed to the preservation of *S. boulardii.* This aligns with the use of Eudragit^®^ S12.5, an anionic copolymer widely recognized for its a enteric coating properties and ability to protect active ingredients from the acidic gastric environment (Ahuja 2024). The selection of two specific weight gains (5% and 10%) for evaluation was based on prior research, which successfully used these percentages to control the release of caffeine from gelatin capsules (Dvorácková et al. 2010). Thus, the capsules coated with a 5% weight gain (using an intermediate layer of hydroxypropyl cellulose to improve the adhesion of Eudragit^®^ S12.5 to gelatin) released caffeine in a pH 6.8 medium, after withstanding two hours in acidic pH. Capsules with a 10% weight gain released their content mainly al pH 7.5, after being exposed to acidic pH for 2 h and to pH 6.8 for 6 h. These results demonstrated the good acid resistance of capsules coated with this polymer and their release under conditions characteristic of the distal ileum.

During the coating processes, stressors such as temperature fluctuations, pH variations, and mechanical friction can compromise viability. Notably, deviations from optimal temperature (e.g., 37 °C) can lead to membrane damage, ionic imbalance, intracellular acidification, reactive oxygen species (ROS) production, and impairment of protein and mitochondrial function (Guimarães et al. 2018).

In our study, the 5% coating was less detrimental to viability of *S. boulardii* than the 10% coating, since this last percentage required longer atomization time and increased exposure to stress factor such as mechanical shear, dehydration, and thermal load during the formation of thicker coatings, which may hinder oxygen diffusion (Patra 2017) and create localized microenvironments with altered humidity or pH gradients during the drying, exacerbating stress (Agriopoulou 2023). These combined effects likely explain the slightly lower viability observed with the 10% coating, even though 10% is the recommended by the manufacturer (Nikam et al. 2023). Furthermore, prolonged coating durations increase exposure to organic solvents or plasticizers (e.g., isopropanol in Eudragit^®^ S12.5), which can potentially disrupt cell membranes and reduce metabolic activity (Cook et al. 2012; D’Amico et al. 2025; Nikam et al. 2023; Phin Yin et al. 2024). While isopropanol can be detrimental at high concentrations, our coating procedure facilitated its rapid evaporation under spray conditions (30–33 °C, 20–25 psi), minimizing direct contact between the liquid solvent and the yeast cells, since the isopropanol was present in the polymer solution phase, not in direct contact with the yeasts suspended in the initial coating (skim milk + HPMC). Viability was systematically assessed after the coating step by plate counting, and no significant reduction in colony-forming units was observed. This confirms that the coating process, including the use of isopropanol, did not compromise *S. boulardii* viability under the conditions employed.

Regarding the percentage of weight variation of the capsules containing pellets coated with 5% and 10% and the disintegration time, both meet the criteria established by the FEUM and European Pharmacopoeia. Particularly, disintegration time in was 40% less than the stablished limit.

Encapsulation efficiency, before and after coating showed minimal viability loss (≤ 0.3 log units). Yields of 100% and 99.61% confirmed successful encapsulation for nearly all pellets. The major viability loss occurred during pellet incorporation (1.5 log), while viability remained stable during coating process, confirming that the coating itself is not detrimental. Overall, this indicates that the encapsulation method preserves viable *S. boulardii* cell effectively.

Compared to previous encapsulation studies, our system performed favorably. It has been reported viability values of 79.67% and 75.82% in simulated gastric and intestinal fluids, respectively, for lyophilized *S. boulardii*. Nevertheless, lower values were observed when encapsulation by ionic gelation was combined with lyophilization (71.42% and 63.49%, respectively) (Poloni et al. 2021). Similarly (Savoldi et al. 2022) reported 79.88 ± 1.35 yield using maltodextrin: rice protein (25:75) in microencapsulation.

Despite, both formulations demonstrated significant enteric protection. In vitro release studies showed that no yeast was released in simulated gastric fluid (pH 1.2), whereas a rapid release was observed upon transfer to alkaline medium. However, full release was not achieved: only 81% and 87% of *S. boulardii* were recovered from 5% to 10% coatings, respectively. This complete release may be due to: (i) incomplete dissolution of Eudragit^®^ S12.5 at pH 7.0, especially in thick or heterogeneously plasticized films (D’Amico et al. 2025; Nikam et al. 2023); (ii) core morphology if yeast cells are embedded within pellet microdomains or hydrocolloid layers (e.g., HPMC), full exposure to the medium may be impaired (Poloni et al. 2021). (iii) yeast adhesion to pellet surfaces, strong interactions between yeast wall components and matrix materials can hinder desorption (Jacobsen et al. 2021; Rodrigues et al. 2020). To better understand these effects, future work should consider extending the dissolution times beyond 20 min under alkaline conditions that simulate the ones found in the intestinal tract; performing quantitative surface analysis (e.g. SEM-EDS) on residual pellets post dissolution; analyzing pellet porosity via imaging techniques such as micro-CT and modeling release kinetics to identify rate limiting mechanism such as diffusion or polymer erosion. Additionally, the impact of digestive enzymes on the covered pellets should be considered, since although Eudragit^®^ S12.5 is specifically designed to resist degradation in acidic gastric conditions and to dissolve at pH values above 7, which are typically encountered in the distal ileum or colon, it is important to acknowledge that once the coating dissolves, the core components, including sugar based excipients and yeast cells, are exposed to the enzymatic environment of the intestine. Digestive enzymes such as pancreatic amylases, proteases, and lipases may degrade the sugar matrix or interact with cell membranes, potentially compromising viability or modifying the release kinetics. However, the use of crystalline sucrose and mannitol in the pellet core, both of which were relatively resistant to rapid enzymatic degradation, contributes to a delayed erosion profile that favors gradual release even after coating dissolution. To substantiate this, in vitro simulation studies are crucial. These studies would allow the assessment of survival rate of *S. boulardii* across sequential exposure to simulated gastric fluid and simulated intestinal fluid containing relevant enzymes such as pepsin, pancreatin, and bile salts. Such models help predict the release kinetics and functional viability under physiological relevant conditions. Incorporating these tests into future experimental designs will provide a more comprehensive understanding of the protective performance and delivery efficiency of the formulation.

Although a direct chemical interaction analysis between the carrier material Eudragit ^®^ S12.5 based coating and the encapsulated *S. boulardii* cells was not performed in this study, the formulation was designed considering well established chemical compatibility. Sugar pellets, composed of sucrose are neutral excipients cores that are widely used in pharmaceutical formulations (Kállai-Szabó et al. 2022). Eudragit^®^ S12.5 is an anionic copolymer with low reactivity under neutral and alkaline pH, serving as a chemically inert protective barrier and is widely used for enteric coatings due to its limited solubility in gastric pH and dissolution above pH 7.0 (Ahuja 2024). Although recent studies on Eudragit^®^ S12.5 with *S. boulardii* specifically are not yet available, analogous investigations with probiotic bacteria using enteric polymers including Eudragit^®^ S12.5 derivatives demonstrating that these coatings preserve cell wall integrity and viability during encapsulation and gastrointestinal transit, without detectable chemical modification of microbial surface components (Pan et al. 2022). In addition, comprehensive reviews between 2023 and 2024 on probiotic encapsulation systems underscore that synthetic enteric polymer often maintain compatibility via passive protection rather than active chemical interaction (Ahuja 2024; Nikam et al. 2023). Encapsulation agents are selected to avoid interactions that could compromise cell viability, focusing on physical encapsulation and pH triggered release rather than bond formation with cellular biomolecules (Phin Yin et al. 2024). Consequently, while we did not perform direct spectroscopic or thermal analyses (e.g., FT-IR, DSC) on Eudragit-cell interactions, the formulation rationale and literature consensus support that Eudragit^®^ S12.5 serves as a chemically inert, protective barrier.

## Conclusion

Thus, this study presents a novel approach for preserving *S. boulardii* viability during manufacturing and simulated gastrointestinal transit by incorporating the yeast into sugar pellets coated with Eudragit^®^ S12.5 enteric polymer. Unlike previous reports that primarily focused on bacterial probiotics and alginate-based matrices, our system successfully applies a layered film coating technique using sugar-based cores and evaluates two concentrations of enteric polymer (5% and 10%). The formulation not only maintained high cell viability (up to 92.6%) throughout the coating process but also demonstrated effective protection under simulated gastric conditions and targeted release in alkaline pH. Compared to conventional methods, the proposed system shows superior performance in terms of encapsulation efficiency, disintegration time, and release kinetics, highlighting its potential for pharmaceutical and biotechnological applications aimed at delivering viable yeast cells to the distal gut.

## Data Availability

The datasets generated and analyzed during the current study are available from the corresponding author upon reasonable request.

## References

[CR1] Agriopoulou S, Tarapoulouzi M, Varzakas T, Jafari SM (2023) Application of encapsulation strategies for probiotics: from individual loading to Co-Encapsulation. Microorganisms, *11*(12) 10.3390/microorganisms11122896 PMC10745938 38138040

[CR2] Ahuja D, Shah A (2024) Eudragit literature: a revolutionary overview. Bull Pure Appl Sciences-Zool, 43

[CR3] Bailon Barceinas KL (2020) Elaboración y evaluación in vitro de Un sistema de liberación modificada a base. de Saccharomyces boulardii

[CR4] Baral KC, Bajracharya R, Lee SH, Han HK (2021) Advancements in the pharmaceutical applications of probiotics: dosage forms and formulation technology. Int J Nanomed 16:7535–7556 10.2147/IJN.S337427 PMC8594788 34795482

[CR5] Blaize JF, Suter E, Corbo CP (2019) Serial dilutions and plating: microbial enumeration

[CR6] Castro-López C, Romero-Luna HE, García HS, Vallejo-Cordoba B, González-Córdova AF, Hernández-Mendoza A (2023) Key stress response mechanisms of probiotics during their journey through the digestive system: a review. Probiotics Antimicrob Proteins 15(5):1250–1270 36001271 10.1007/s12602-022-09981-x

[CR7] Chen J, Chen X, Ho CL (2021) Recent development of probiotic bifidobacteria for treating human Diseases. [Review]. Front Bioeng Biotechnol, *9* 10.3389/fbioe.2021.770248 PMC8727868 35004640

[CR8] Cook MT, Tzortzis G, Charalampopoulos D, Khutoryanskiy VV (2012) Microencapsulation of probiotics for gastrointestinal delivery. J Control Release 162(1):56–67 22698940 10.1016/j.jconrel.2012.06.003

[CR9] D’Amico V, Cavaliere M, Ivone M, Lacassia C, Celano G, Vacca M et al (2025) Microencapsulation of probiotics for enhanced stability and health benefits in dairy functional foods: a focus on pasta filata cheese. Pharmaceutics. 10.3390/pharmaceutics17020185 40006552 10.3390/pharmaceutics17020185 PMC11859715

[CR10] de Carvalho Bruna T, Subotić A, Vandecruys P, Deleu S, Vermeire S, Johan T, M (2024) Enhancing probiotic impact: engineering *Saccharomyces boulardii* for optimal acetic acid production and gastric passage tolerance. Appl Environ Microbiol 90(6):e00325–e00324 38752748 10.1128/aem.00325-24 PMC11218656

[CR11] De Simone C (2019) The unregulated probiotic market. Clin Gastroenterol Hepatol 17(5):809–817 29378309 10.1016/j.cgh.2018.01.018

[CR12] Dvorácková K, Rabisková M, Gajdziok J, Vetchý D, Muselík J, Bernatoniene J et al (2010) Coated capsules for drug targeting to proximal and distal part of human intestine. Acta Pol Pharm 67(2):191–199 20369797

[CR13] Erdélyi L, Fenyvesi F, Gál B, Haimhoffer Á, Vasvári G, Budai I (2022) Investigation of the role and effectiveness of chitosan coating on probiotic microcapsules. Polymers. 10.3390/polym14091664 35566837 10.3390/polym14091664 PMC9101405

[CR14] Ganje M, Sekhavatizadeh SS, Teymouri F, Gilkheiri M, Rahmani B (2024) Encapsulation of *Lacticaseibacillus rhamnosus* by extrusion method to access the viability in saffron milk dessert and under simulated gastrointestinal conditions. Food Sci Nutr 12(11):9714–9726 39619975 10.1002/fsn3.4510 PMC11606897

[CR15] Guimarães A, Abrunhosa L, Pastrana LM, Cerqueira MA (2018) Edible films and coatings as carriers of living microorganisms: a new strategy towards biopreservation and healthier foods. Compr Rev Food Sci Food Saf 17(3):594–614 33350124 10.1111/1541-4337.12345

[CR16] How Y-H, Yeo S-K (2021) Oral probiotic and its delivery carriers to improve oral health: a review. Microbiology 167(8):001076 10.1099/mic.0.001076 34351255

[CR17] Jacobsen NMY, Nedergaard HB, Kock A, Caglayan I, Laursen MM, Lange E-M et al (2021) Development of gastro-resistant coated probiotic granulates and evaluation of viability and release during simulated upper gastrointestinal transit. LWT 144:111174

[CR18] Kelesidis T, Pothoulakis C (2012) Efficacy and safety of the probiotic *Saccharomyces boulardii* for the prevention and therapy of gastrointestinal disorders. Ther Adv Gastroenterol 5(2):111–125 10.1177/1756283X11428502 PMC3296087 22423260

[CR19] Kállai-Szabó N, Lengyel M, Farkas D, Barna Á, Fleck T, Basa C, B., et al (2022) Review on starter pellets: inert and functional cores. Pharmaceutics, 14(6) 10.3390/pharmaceutics14061299 PMC9227027 35745872

[CR20] Kandur B, UĞUrlu T, Rayaman E, ŞAhbaz S (2024) Oral pharmabiotic tablet formulations. J Res Pharm, 28(1)

[CR21] Latif A, Shehzad A, Niazi S, Zahid A, Ashraf W, Iqbal MW et al (2023) Probiotics: mechanism of action, health benefits and their application in food industries. Front Microbiol 14:1216674 37664108 10.3389/fmicb.2023.1216674 PMC10470842

[CR22] Yus C, Gracia R, Larrea A, Andreu V, Irusta S, Sebastian V et al (2019) Targeted release of probiotics from enteric microparticulated formulations. Polymers. 10.3390/polym11101668 31614915 10.3390/polym11101668 PMC6835770

[CR23] Łętocha A, Michalczyk A, Miastkowska M, Sikora E (2024) Effect of encapsulation of *Lactobacillus casei* in alginate–tapioca flour microspheres coated with different biopolymers on the viability of probiotic bacteria. ACS Appl Mater Interfaces 16(39):52878–52893 39301782 10.1021/acsami.4c10187 PMC11450766

[CR24] Liu C, Ma N, Feng Y, Zhou M, Li H, Zhang X et al (2023) From probiotics to postbiotics: concepts and applications. Animal Research and One Health 1(1):92–114

[CR25] Majeed SM, Al-Shaheen MK, Al-Zidan RN, Mahmood SM (2020) Multiple unite pellet systems (MUPS) as drug delivery model. J Drug Deliv Ther 10(6):231–235

[CR26] Mudgil P, Alkaabi F, Khan H, Javed M, Hajamohideen AR, Hamed F et al (2024) Enhanced viability and stability of the *Lactobacillus reuteri* DSM 17938 probiotic strain following microencapsulation in pea and rice protein-inulin conjugates. [Original Research]. Front Sustain Food Syst 8–2024

[CR27] Nikam A, Sahoo PR, Musale S, Pagar RR, Paiva-Santos AC, Giram PS (2023) A systematic overview of Eudragit^®^ based copolymer for smart healthcare. Pharmaceutics 15(2):587 36839910 10.3390/pharmaceutics15020587 PMC9962897

[CR28] Pan J, Gong G, Wang Q, Shang J, He Y, Catania C et al (2022) A single-cell nanocoating of probiotics for enhanced amelioration of antibiotic-associated diarrhea. Nat Commun 13:2117 35440537 10.1038/s41467-022-29672-z PMC9019008

[CR29] Patra C, Priya R, Swain SK, Jena G, Panigrahi K, Ghose D (2017) Pharmaceutical significance of eudragit: A review. Future J Pharm Sci, *3*

[CR30] Phin Yin S, Hian T, Asras M, Karmawan L (2024) From preparation to product: factors influencing probiotic viability in spray drying. Curr Sci Technol 4:22–35

[CR31] Poloni VL, Bainotti MB, Vergara LD, Escobar F, Montenegro M, Cavaglieri L (2021) Influence of technological procedures on viability, probiotic and anti-mycotoxin properties of *Saccharomyces boulardii* RC009, and biological safety studies. Curr Res Food Sci 4:132–140 33778773 10.1016/j.crfs.2021.02.006 PMC7985476

[CR32] Razavi S, Janfaza S, Tasnim N, Gibson DL, Hoorfar M (2021) Nanomaterial-based encapsulation for controlled gastrointestinal delivery of viable probiotic bacteria. Nanoscale Adv 3(10):2699–2709 36134186 10.1039/d0na00952k PMC9419840

[CR33] Rodrigues FJ, Cedran MF, Bicas JL, Sato HH (2020) Encapsulated probiotic cells: relevant techniques, natural sources as encapsulating materials and food applications–a narrative review. Food Res Int 137:109682 33233258 10.1016/j.foodres.2020.109682

[CR34] Sionek B, Szydłowska A, Trząskowska M, Kołożyn-Krajewska D (2024) The impact of physicochemical conditions on lactic acid bacteria survival in food products. Fermentation 10(6):298

[CR35] Roy S, Dhaneshwar S (2023) Role of prebiotics, probiotics, and synbiotics in management of inflammatory bowel disease: current perspectives. World J Gastroenterol 29(14):2078–2100 37122604 10.3748/wjg.v29.i14.2078 PMC10130969

[CR36] Sánchez-Portilla Z, Melgoza-Contreras LM, Reynoso-Camacho R, Pérez-Carreón JI, Gutiérrez-Nava A (2020) Incorporation of *Bifidobacterium* sp. into powder products through a fluidized bed process for enteric targeted release. J Dairy Sci 103(12):11129–11137 33069409 10.3168/jds.2020-18516

[CR37] Savoldi TE, Scheufele FB, Drunkler DA, da Silva GJ, de Lima JD, Maestre KL et al (2022) Microencapsulation of *Saccharomyces boulardii* using vegan and vegetarian wall materials. J Food Process Preserv 46(7):e16596

[CR38] Suez J, Zmora N, Segal E, Elinav E (2019) The pros, cons, and many unknowns of probiotics. Nat Med 25(5):716–729 31061539 10.1038/s41591-019-0439-x

[CR39] Thinkohkaew K, Jonjaroen V, Niamsiri N, Panya A, Suppavorasatit I, Potiyaraj P (2024) Microencapsulation of probiotics in chitosan-coated alginate/gellan gum: optimization for viability and stability enhancement. Food Hydrocolloids 151:109788

[CR40] Tung JM, Dolovich LR, Lee CH (2009) Prevention of *clostridium difficile* infection with *Saccharomyces boulardii*: a systematic review. Can J Gastroenterol 23(12):817–821 20011734 10.1155/2009/915847 PMC2805518

[CR41] Vergkizi S, Partheniadis I, Sipaki A, Papanikolaou T, Fatouros D, Nikolakakis I (2024) Hydroxyapatite and pore former effects on the microstructure and mechanical strength of porous pellets loaded with *Lactobacillus*. Powder Technol 435:119433

[CR42] Wang A, Zhong Q (2024) Drying of probiotics to enhance the viability during preparation, storage, food application, and digestion: a review. Compr Rev Food Sci Food Saf 23(1):e13287 38284583 10.1111/1541-4337.13287

[CR43] Wang G, Chen Y, Xia Y, Song X, Ai L (2022) Characteristics of probiotic preparations and their applications. Foods 11(16):2472 36010472 10.3390/foods11162472 PMC9407510

[CR44] Xu Y, Zhu S, Sun X, Shan K, Zhang C, Xiao H et al (2025) Fabrication of microcapsules encapsulating *L. rhamnosus* GG with Eudragit^®^ L100–trehalose and polysaccharides: a study on physicochemical properties and cell adhesion. Sustainable Food Technology 3(4):1064–1073. 10.1039/D5FB00084J

